# *Calophyllum brasiliense* Modulates the Immune Response and Promotes *Leishmania amazonensis* Intracellular Death

**DOI:** 10.1155/2018/6148351

**Published:** 2018-02-13

**Authors:** L. Domeneghetti, I. G. Demarchi, J. Z. Caitano, R. B. Pedroso, T. G. V. Silveira, M. V. C. Lonardoni

**Affiliations:** ^1^Graduate Program in Bioscience and Physiopathology, Departamento de Análises Clínicas e Biomedicina, The State University of Maringá, Colombo Avenue 5790, 87020-900 Maringá, PR, Brazil; ^2^Departamento de Análises Clínicas e Biomedicina, The State University of Maringá, Maringá, PR, Brazil; ^3^Postgraduate Program in Health Sciences, Centro de Ciências da Saúde, State University of Maringá, Maringá, PR, Brazil

## Abstract

*Calophyllum brasiliense* is a plant from the Brazilian rain forests and has been used in folk medicine for the treatment of various diseases, including leishmaniasis. This infectious disease depends on the *Leishmania* sp. and the host immune response. *C. brasiliense* antileishmanial activity is well known, but the effects on immune response remain to be investigated. This study showed the leishmanicidal and immunomodulatory effects of a 30 *μ*g/mL of hydroalcoholic extract of *C. brasiliense* in murine macrophages before and after *Leishmania* (*Leishmania*) *amazonensis* infection. The semiquantitative cytokine RNA expression was determined by RT-PCR and the anti-*Leishmania* activity was measured by infection index (IF). Hydroalcoholic extract of *C. brasiliense* reduced more than 95% of IF when used before and after *Leishmania* infection, with 3 and 24 h of treatment (*p* < 0.05). *C. brasiliense* inhibited or reduced significantly (*p* < 0.05) the TNF-*α*, IL-1*β*, IL-18, and IL-10 mRNA expression. The antileishmanial and anti-inflammatory effects showed the potential of *C. brasiliense* as an alternative therapy for leishmaniasis and it must be investigated.

## 1. Introduction

Leishmaniases are endemic neglected tropical diseases caused by intracellular parasitic protozoa of the genus *Leishmania*. The parasite has a complex digenetic life cycle requiring a susceptible vertebrate host and an insect vector, which allow their transmission. *Leishmania* promastigote forms are inoculated by the phlebotomy vector in the skin of the host. The parasites invade macrophage cells transforming them into amastigote form [1, 2]. The initial events involve the invasion of the host cells by *Leishmania* and their transport to the regional lymph nodes, in which a microenvironment is created from immune response and parasite [1–3]. The clinical manifestations depend on complex interactions between the parasite species and the host immune system, which result in different clinical forms of cutaneous, mucocutaneous, diffuse, or visceral diseases [1, 2].

The infection caused by *Leishmania* (*Leishmania*) *amazonensis* is characterized by skin and diffuse lesions, and the immune response of the host is essential for the resolution of leishmaniasis. The immune mechanisms involved will define the fate of the infection and its spread or control, as well as the development of the adaptive immune response [3, 4]. *Leishmania* parasites are recognized by immune cells stimulating the T helper 1 lymphocytes (Th1) to produce cytokines that induce the activation of the phagocytic mononuclear system. The macrophage cells produce the microbicidal substances leading to parasite death. The reactive oxygen species (ROS) and oxide nitric are microbicidal agents that promote the death of *Leishmania* sp. [4]. The Th1 immune response is related to clinic cure and therapeutic success [3, 4].

Currently, leishmaniasis has affected 12 million people worldwide, occurring 70,000 deaths every year and there are 350 million people at risk of contracting the disease [5]. Leishmaniasis treatment is based on Glucantime or amphotericin B, but these drugs have shown severe adverse effects, potential toxicity, and therapeutic failure, which lead to the abandonment of the therapy or even its ineffectiveness [5]. Furthermore, antileishmaniasis products that are extracted from nature have increasingly been patented each year, demonstrating the importance of bioprospecting studies to improve the armamentarium of antileishmaniasis drugs [6].

Plants are traditionally used for the treatment of many diseases and are more accessible, as they can be naturally acquired [7–9], leading them to the position of potential candidates for leishmaniasis treatment [10–13]. *Calophyllum brasiliense* Camb. (Clusiaceae) is a tree popularly known as “guanadi-cedar,” “oil stick,” and “guarandi” [13, 14] and it has extensive natural distribution, on rain forests on wet and marshy soils [15], occurring from Mexico to the south coast of Brazil [16]. The extracts and derivatives from *C. brasiliense* leaves have been used in folk medicine against various diseases [9–13, 17–27], including leishmaniasis [10–13]. Some biological activities of *C. brasiliense* were reported such as its antibacterial, antifungal, and antiprotozoal activities, its cytotoxicity to tumor cells, and its inhibitory effect on tumor promotion and HIV-1 replication [10–13, 19–27]. The leishmanicidal activity of *C. brasiliense* is known [10–13], but the other immunomodulatory effects of *C. brasiliense* remain unclear [20].

Blanco-Ayala and coworkers conducted an investigation suggesting that the xanthones from *C. brasiliense* played a role as potential agents to attenuate the oxidative damage produced by different prooxidants, such as ROS and lipid peroxidation [20]. ROS and nitric oxide are essential to parasite death [2]. Besides that, *L.* (*L.*) *amazonensis* can escape and survive at the immune environment, requiring the modulation of the components of the immune response as Th1 and Th2 cells and their products [2, 28], but its association with *C. brasiliense* treatment remains to be investigated. Therefore, this study investigated the potential of hydroalcoholic extract of *C. brasiliense* in immunomodulating the cytokines' expression by murine macrophages infected with *L.* (*L.*) *amazonensis*.

## 2. Materials and Methods

### 2.1. Culture and Maintenance of Parasite

Promastigotes forms of *L.* (*L.*) *amazonensis* (MHOM/BR/1977/LTB0016), held in cryopreservation, were thawed and maintained at 25°C in 199 culture medium supplemented with fetal bovine serum 10% (*v*/*v*), human urine (1%), L-glutamine (20 mM), and antibiotics (penicillin 100 IU/ml and streptomycin 0.1 mg/ml). Weekly, subcultures were made of 199 medium cultures and parasite inoculum was adjusted according to each experiment.

### 2.2. Animals

BALB/c isogenic mice, female, 8–10 weeks, were obtained from the Central Animal Laboratory at the State University of Maringá, after approval by the Ethics Committee on the Use of Experimental Animals of the State University of Maringá (Paraná State, Brazil) (warrant number 133/2012). The animals were kept in collective cages with water and food *ad libitum* with 12 h light-dark cycles.

### 2.3. Plant Material

The leaves of *C. brasiliense* were collected between January 2009 to December 2010, in Parque Estadual da Ilha do Cardoso in Cananéia, located on the southern coast of São Paulo state (Brazil). This tree is found in all coastal Atlantic forest vegetation (https://nossosparques.org.br/). The plant was identified by Dr. Maria CM Young, and a voucher specimen was deposited (SP 363818) in the herbarium of the Botanical Institute of São Paulo, SP, Brazil. The plant sample was weighed, dried in a circulating air oven (Quimiss Q-31, Diadema, SP, Brazil) at 35°C, crushed in a Wiley mill (Tecnal Marconis, TE048), and subsequently stored in a dry place, away from light.

### 2.4. Hydroalcoholic Extract of *C. brasiliense*

The sample (1.3 kg), previously crushed, underwent cold maceration process in ethanol : water (9 : 1) and successive extractions until all active compounds were retrieved (4 × 61) [29]. Subsequently, the extract was filtered and vacuum evaporated at 35–40°C on a rotating evaporator until total elimination of the organic solvent. The product of this evaporation, a dark green water-insoluble residue, was dissolved in dichloromethane. The organic solvent was then removed by evaporation at room temperature, and the obtained hydroalcoholic extract (≈75.0 g) was stored at −10°C, protected from light until use [10].

### 2.5. Cell Culture and Treatments

Peritoneal macrophages from BALB/c mice were collected with sterile cold RPMI 1640 medium (pH 7.6) and 1.0 × 10^6^ macrophages/mL (in RPMI 1640 supplemented with 100 U/ml penicillin, 0.1 mg/mL streptomycin, and 0.05 mg/ml gentamycin) were plated on twelve-well culture plates and incubated at 37°C in an atmosphere of 5% CO_2_ for one hour. Subsequently, the nonadherent cells were removed, and the adherent cells were infected with 10 promastigotes of *L.* (*L.*) *amazonensis* by macrophage and incubated for 3 h at 37°C with 5% CO_2_. Hence, cultures were treated with 30 *μ*g/mL of the hydroalcoholic extract of *C. brasiliense* for 3 h [13]. Alternatively, cultures were pretreated with 30 *μ*g/mL of the hydroalcoholic extract of *C. brasiliense* for 3 h and then infected with 10 promastigotes per macrophage, for 3 h under the conditions mentioned above. Noninfected macrophages were also treated with 30 *μ*g/mL of hydroalcoholic extract of *C. brasiliense* for 3 h. Macrophages infected with promastigotes of *L.* (*L.*) *amazonensis* and untreated were used as positive control, and macrophage uninfected and untreated were used as negative control. All procedures were performed in triplicate.

### 2.6. Antiamastigote Activity (Infection Index)

A suspension of 5 × 10^5^ macrophages/mL was added on 13 mm-diameter sterile glass coverslips (Glastecnica, São Paulo, SP, Brazil), distributed into 24-well culture plates and then incubated at 37°C with an atmosphere containing 5% CO_2_ for 1 h. After that, nonadherent cells were removed by washing with RPMI 1640 medium, while the adherent cells were infected with promastigotes of *L.* (*L.*) *amazonensis* (six parasites per macrophage) [30], and incubated for 3 h. Then, the supernatant was discarded, and the infected cells were treated with 30 *μ*g/mL of hydroalcoholic extract of *C. brasiliense* for 3 to 24 h. In another condition, macrophages were first treated for 3 h with 30 *μ*g/mL hydroalcoholic extract of *C. brasiliense*, and after this period, the cells were infected with promastigotes of *L.* (*L.*) *amazonensis* (six parasites per macrophage) incubated for 3 to 24 hours. After these periods of treatment, the supernatant was discarded and the coverslips removed, washed, and stained with Panoptic kit Quick LB® (Laborclin, Curitiba, PR, Brazil), and after, it is fixed on glass slides (24 × 76 mm) with Entellan (Merck, Darmstadt, Germany). The experiments were performed three times in triplicate. The macrophages infected with *L.* (*L.*) *amazonensis* and untreated were used as positive control and macrophages uninfected and untreated as negative control. The antiamastigote activity of *C. brasiliense* was determined by the infection index (IF) calculated by the percentage of infected macrophages multiplied by the average number of parasites per macrophage.

### 2.7. mRNA Analysis by Semiquantitative Reverse Transcriptase-Polymerase Chain Reaction (RT-PCR)

Total RNA was extracted from macrophages using TRIzol reagent according to the manufacturer's instructions (Invitrogen, Saint Louis, USA). The complementary DNA (cDNA) was synthesized from 1.0 *μ*g of total RNA as a template in a reverse transcription reaction using Superscript III reverse transcriptase (Invitrogen, Carlsbad, CA, USA). RNA quantification (ng/ml) and the determination of purity were performed using NANODROP 2000 UV-Vis Spectrophotometer (Thermo Fisher Scientific Inc., USA) (optimal purity level = 1.8). cDNA samples were amplified by PCR using the specific primer sequences for mRNA: tumor necrosis factor (TNF-*α*) [31], interleukin (IL) 1*β* [32], IL-12, IL-18 [31], interferon-*γ* (IFN-*γ*) [32], IL-10, enzyme nitric oxide synthase (iNOS) [31], and glyceraldehyde-3 phosphate dehydrogenase (GAPDH and mRNA; *Mus musculus*) as internal control of the PCR amplification [33] (Table 1). The primers were chosen according to the BLAST tool that is available in the GenBank database and previous studies. The reaction mixture contained 25 *μ*M of each primer, 0.2 mM dNTP MIX, 1 U Platinum Taq DNA polymerase (Invitrogen), and 1.5 mM MgCl_2_, and DNA (5–10 *μ*L~100 pg/reaction) was added to each test tube in a final volume of 25 *μ*L. The amplified DNA fragments were separated by 1.5% agarose gel electrophoresis and revealed with ethidium bromide in transilluminator. Semiquantitative RT-PCR was performed by quantifying the bands densitometrically using ImageJ software (National Institutes of Health, Bethesda, MD, USA). Each cytokine gene was normalized to GAPDH as a housekeeping gene control (internal control).

### 2.8. Statistical Analysis

The results were tested for normality of distributions and the data subsequently analyzed by Mann–Whitney *U* test using STATISTICA 7.0 software and *p* < 0.05 is considered significant.

## 3. Results and Discussion

**Figure 1:**
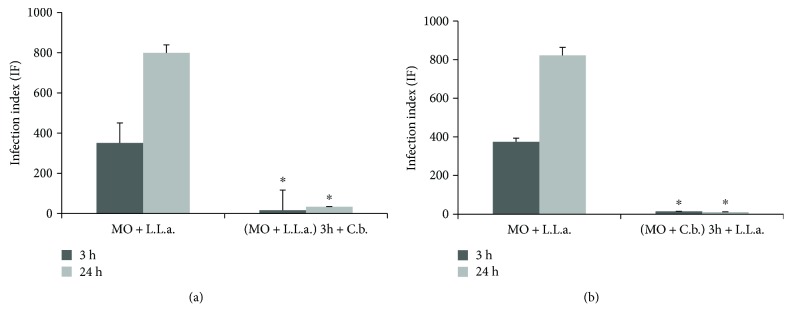
Leishmanicidal activity of *Calophyllum brasiliense*: 5 × 10^5^ macrophages/mL on glass coverslips were (a) infected with *L.* (L.) *amazonensis* promastigotes (6 parasites per macrophage) during 3 h and treated with hydroalcoholic extract of *C. brasiliense* (30 *μ*g/mL) and infection index was obtained after 3 and 24 h of treatment. (b) Cultures were treated with *C. brasiliense* alcoholic extract (30 *μ*g/mL) for 3 h then they were infected with *L.* (*L.*) *amazonensis*, and the infection index was obtained after 3 and 24 hours. Macrophages infected with *L.* (*L.*) *amazonensis* and without treatment were controls. ^∗^*p* < 0.05 compared to controls.

*C. brasiliense* plant is a folk medicine used in many infections, such as leishmaniasis [10–13], in Brazil and other countries [9–13, 17–27]. In the present study, we showed anti-*Leishmania* activity and immunomodulatory effects of *C. brasiliense* during infection. Murine macrophages infected with *L.* (*L.*) *amazonensis* and treated with 30 *μ*g/mL hydroalcoholic extract of *C. brasiliense* showed a significant reduction in the infection index (IF) (*p* < 0.05) after 3 h (IF: 16.5, 95.3% of reduction) and after 24 h (IF: 33.0, 95.9% of reduction), when compared to untreated cells (IF: 351/3 h and 799/24 h) (Figure 1(a)). The leishmanicidal results are similar to those of Brenzan et al. [11], who related the leishmanicidal activity of *C. brasiliense* for promastigote and axenic amastigote *L.* (*L.*) *amazonensis* in a concentration between 20 and 40 *μ*g/mL and did not observe cytotoxic activity.

In subsequent experiments, macrophages were pretreated with *C. brasiliense* extract and infected with *L.* (*L.*) *amazonensis* for 3 and 24 h (Figure 1(b)). The IF was also significantly reduced (*p* < 0.05) compared to controls, from 374.5 to 15 (96%) after 3 h and from 822.3 to 11.6 (98.6%) after 24 h.

Honda et al. [13] observed that pretreatment with dichloromethane extract (10%) or with hexane fraction (5%) of experimentally infected BALB/c mice decreased the volume of the lesions by *L.* (*L.*) *amazonensis*. The animals treated topically during 32 days after the infection revealed healing lesions, and the parasite load in the popliteal lymph nodes was significantly reduced in treated animals. The results showed that crude extract and hexane fraction of *C. brasiliense* have a significant *in vitro* and *in vivo* leishmanicidal activity [13]. These studies indicate the vegetal species' therapeutic potential in obtaining phytotherapeutic drugs [10–13].

The compounds derivate from *C. brasiliense*, such as (−) mammea A/BB and 5,7-dihydroxy-8-(2-methylbutanoyl)-6-(3-methylbutyl)-4-phenyl-chroman-2-one, may have antileishmanial activity by acting directly on the depolarization of the *Leishmania* mitochondria membrane potential, thus leading to parasite death [12]. A topical formulation containing *C. brasiliense* Camb. extract promoted wound healing in rats with cutaneous leishmaniasis; after 14 days of the treatment start, the animals treated exhibited more than 90% reduction of wound areas. After 21 days of treatment, the treated animals exhibited a significant increase in fibroblasts. Thus, the *C. brasiliense* emulsion had healing properties in the topical treatment of wounds and accelerated the healing process [13].

**Figure 2:**
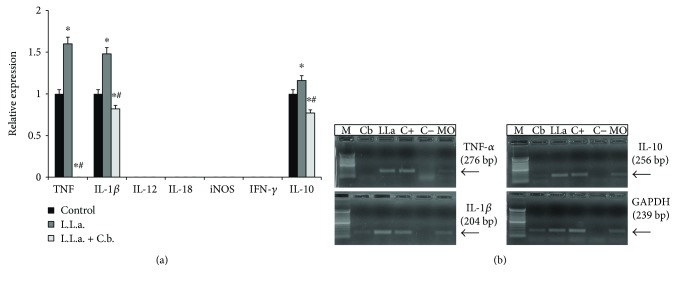
Expression levels of cytokines and iNOS mRNA by macrophages infected with *L.* (*L.*) *amazonensis* or treated with hydroalcoholic extract of *C. brasiliense*. (a) L.L.a: *L.* (*L.*) *amazonensis*; C.b.: *C. brasiliense*. L.L.a + C.b.: peritoneal macrophages from BALB/c mice were infected with promastigotes of *L.* (*L.*) *amazonensis* (10 parasites : 1 macrophage) for 3 h and then treated with hydroalcoholic extract of *C. brasiliense* (30 *μ*g/ml) for 3 h; L.L.a.: macrophages infected and untreated; control: macrophages uninfected and untreated were incubated only with RPMI during 6 h (negative control) ^∗^*p* < 0.05 significantly compared with negative control; ^#^*p* < 0.05 compared to infected macrophages. (b) In 1.5% agarose gel electrophoresis, the products of reverse transcriptase-polymerase chain reaction for conditions above were revealed with ethidium bromide in a transilluminator. M: molecular marker; C−: amplification negative control (H_2_O); C+: DNA obtained from macrophages stimulated with 5 *μ*g/mL of lipopolysaccharide (LPS); MO: macrophages (negative control, uninfected and untreated); Cb: macrophages treated with *C. brasiliense*; LLa: macrophages infected with *L.* (*L.*) *amazonensis*.

**Figure 3:**
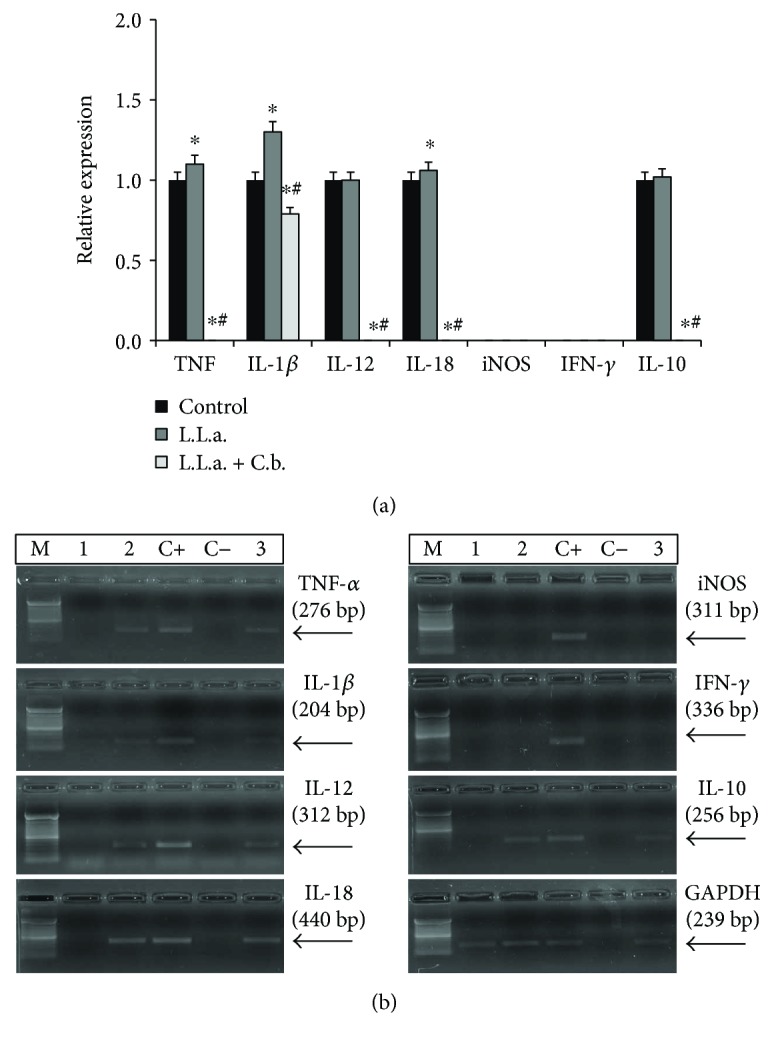
Expression levels of cytokine and iNOS mRNA by macrophages infected with *L.* (*L.*) *amazonensis* and treated with hydroalcoholic extract of *C. brasiliense*. (a) L.L.a: *L.* (*L.*) *amazonensis*; C.b.: *C. brasiliense*. L.L.a + C.b.: peritoneal macrophages from BALB/c mice were infected with promastigotes of *L.* (*L.*) *amazonensis* (10 parasites: 1 macrophage) for 3 h and then treated with hydroalcoholic extract of *C. brasiliense* (30 *μ*g/ml) for 3 h; L.L.a.: macrophages infected and untreated; control: macrophages uninfected and untreated were incubated only with RPMI during 6 h (negative control). ^∗^*p* < 0.05 significantly compared to negative control; ^#^*p* < 0.05 compared to infected and untreated macrophages. (b) Cytokine mRNA expression by semiquantitative reverse transcriptase-polymerase chain reaction (RT-PCR) DNA fragments was separated in 1.5% agarose gel electrophoresis and revealed with ethidium bromide in a transilluminator. M: molecular marker; C−: amplification negative control (H_2_O); C+: DNA obtained from macrophages stimulated with 5 *μ*g/mL of LPS (positive control); 1 and MO: macrophages (negative control, uninfected and untreated); 2 and L.L.a. + C.b.: macrophages infected with *L.* (*L.*) *amazonensis* and treated with *C. brasiliense*; 3 and L.L.a.: macrophages infected with *L.* (*L.*) *amazonensis*.

**Figure 4:**
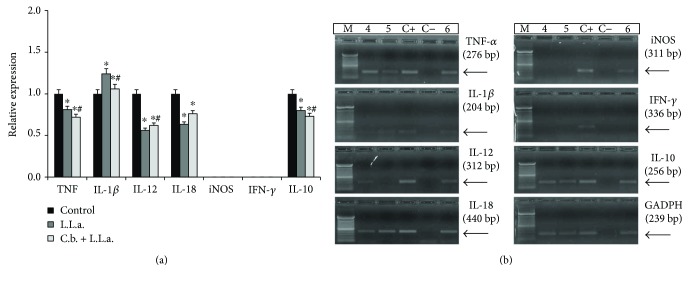
Expression levels of cytokine and iNOS mRNA by macrophages treated with hydroalcoholic extract of *C. brasiliense* and infected with *Leishmania* (*L.*) *amazonensis*. (a) L.L.a.: *L.* (*L.*) *amazonensis*; C.b.: *C. brasiliense*. Peritoneal macrophages from BALB/c mice were treated with hydroalcoholic extract of *C. brasiliense* (30 *μ*g/mL) for 3 h and infected with promastigotes of *L.* (*L.*) *amazonensis* (10 parasites : 1 macrophage) by more than 3 h. Macrophages were only infected with promastigotes of *L.* (*L.*) *amazonensis* (10 : 1) and untreated. As a negative control, macrophages were incubated only with RPMI for 6 h. ^∗^*p* < 0.05 significantly compared to negative control; ^#^*p* < 0.05 compared to infected and untreated macrophages. (b) Cytokine mRNA expression by semiquantitative reverse transcriptase-polymerase chain reaction (RT-PCR) DNA fragments was separated in 1.5% agarose gel electrophoresis and revealed with ethidium bromide in a transilluminator. M: molecular marker; C−: amplification negative control (H_2_O); C+: DNA obtained from macrophages stimulated with 5 *μ*g/mL of LPS; MO: macrophages (negative control, uninfected and untreated); 4 and C.b. + L.L.a.: macrophages treated with *C. brasiliense* for 3 h and infected with *L.* (*L.*) *amazonenis*; 5 and L.L.a.: macrophages infected with *L.* (*L.*) *amazonensis*; 6: macrophages uninfected and untreated.

Besides antileishmanial activity, researches have been conducted to discover new drugs with lower toxicity and immunomodulatory effects for leishmaniasis therapy [28, 34]. *C. brasiliense* treatment modulated the mRNA cytokine expression produced by murine macrophages infected with *Leishmania* and did not modify iNOS mRNA expression. From all cytokines investigated, *C. brasiliense* inhibited TNF-*α* mRNA expression (100%) and reduced in 18% IL-1*β* and 23% IL-10 by uninfected macrophages, compared to untreated and uninfected macrophages (negative control) (*p* < 0.05) (Figure 2). The infection increased 60% of TNF-*α* mRNA expression, IL-1*β* (48%), and IL-10 by macrophages, compared to the negative control (*p* < 0.05) (Figure 2). TNF-*α*, IL-1*β*, and IL-10 expression by infected and treated macrophages was significantly lower than infected macrophages (*p* < 0.05, Figure 2). Macrophages (negative control) did not express iNOS and IFN-*γ* mRNA in all period (Figures 2-4) and IL-12 and IL-18 in 3 h (Figure 2).

In infected and after treated with *C. brasiliense* macrophages, the hydroalcoholic extract inhibited 100% mRNA expression of TNF-*α*, IL-10, IL-12, and IL-18 and reduced 51% IL-1*β*, compared to the positive control (Figure 3). *L.* (*L.*) *amazonensis* infection induced an increase of TNF-*α* (10%), IL-1*β* (30%), and IL-18 (6%) expression, in comparison to the negative control (Figure 3). The *C. brasiliense* treatment subverted the cytokine expression induced by infection since it inhibited TNF-*α*, IL-12, IL-18, and IL-10 expression and reduced the IL-1*β* expression after infection and treatment (Figure 3).

In macrophages pretreated with *C. brasiliense* and after infected with *Leishmania*, the hydroalcoholic extract reduced TNF-*α* expression (9%), IL-12 (6%), IL-18 (15%), and IL-10 (7%) and increased IL-1*β*, compared to the negative control (Figure 4) (*p* < 0.05). The parasite infection promoted the increase of IL-1*β* and reduced TNF-*α*, IL-12, IL-18, and IL-10, compared to the negative control (Figure 4). The previous *C. brasiliense* treatment reduced significantly these same cytokines (*p* < 0.05) even after the infection and increased IL-12 (Figure 4), but it did not influence IL-18 expression.

*C. brasiliense* subverted the rise of mRNA expression of these cytokines (TNF-*α*, IL-1*β*, IL-18, and IL-10) when used after (Figure 3) or before the *Leishmania* infection (Figure 4). Thus, in the present study, *C. brasiliense* modulated the cytokines mRNA expression by murine macrophages when infected or not with *Leishmania*, and, when *C. brasiliense* was tested after and before the infection, it showed the potential of this plant for leishmaniasis treatment. The modulation of cytokines is one of the defense strategies of the host immune system against parasitic infections. In the immune response to *Leishmania* spp., there is an initial activation of the monocyte-macrophage system through cytokines [2].

Interactions between parasite and immune cells lead to phagocytosis by macrophages that activate trigger mechanisms for their microbicidal response to the production of reactive oxygen metabolites (ROS) and nitric oxide (NO) [2]. As the infection progresses, amastigotes multiply within macrophages, which can present parasite antigens to T and B lymphocytes and these respond by producing specific Th, antibodies, cytokines, and other mediators which, in turn, can activate macrophages to destroy the intracellular *Leishmania* [35]. The NO is a main leishmanicidal agent produced by the host, and its production depends on iNOS expression [2]. In *Leishmania* (*L.*) *amazonensis* infection, some researchers related that this parasite species can decrease the production of nitric oxide as an escape mechanism of the immune host [2, 28, 36]. In this study, the iNOS mRNA was not modulated by *C. brasiliense* during infection, but other studies showed that *C. brasiliense* can play a role as a potential agent to attenuate the oxidative damage produced by different prooxidants, as ROS and lipid peroxidation [20]. Although the *C. brasiliense* was not able to modulate iNOS, other immune mechanisms can be investigated, like the cytokines.

Cytokines are protein molecules released by various cell types of the immune system and act on the emission of signals between the cells for immune responses. Several studies in murine models have associated a specific cytokine or a combination of cytokines as factors to susceptibility or resistance to *Leishmania* infection. In this context, a protective immunity for leishmaniasis is related to an efficient Th1 cellular response and the production of cytokines such as IL-12 and IFN-*γ* [37, 38]. In this study, IFN-*γ* was not modulated by *C. brasiliense*, and only IL-12 was increased when *C. brasiliense* was tested before the infection. IL-12 is involved in cell-mediated immunity by stimulating Th1 cells and inflammation control, and it is critical for the dissemination of parasite control [2].

Also, TNF-*α* expression levels were elevated in macrophages infected by *L.* (*L.*) *amazonensis*, but *C. brasiliense* was able to subvert it. Another study reported a relationship between increases in TNF-*α* and the susceptibility to *L.* (*L.*) *amazonensis* infection, inducing the persistent parasitic infection and tissue damage [39]. TNF-*α* is a proinflammatory cytokine of the Th1 cellular response involved in the occurrence of an intense inflammatory reaction and damage to these tissues and results in the appearance of skin and mucosal ulcers [40]. In the present study, *C. brasiliense* inhibited the TNF-*α* mRNA expression completely when used alone or after the *Leishmania* infection, and it was reduced it when used before *Leishmania* infection. These results showed a potential of *C. brasiliense* to reduce the inflammatory cytokines that can lead to the worsening of the leishmaniasis lesions.

*C. brasiliense* also inhibited IL-18 mRNA expression in infected macrophages. This cytokine is primarily produced by macrophages and dendrites cells and is considered as a cofactor for the IL-12-induced development of the Th1 immune response, which optimizes IFN-*γ* production by effector Th1 cells [41]. IL-18 inhibition caused by *C. brasiliense* treatment can explain the IL-12 and IFN-*γ* inhibition. Thus, hydroalcoholic extract of *C. brasiliense* has shown potential anti-inflammatory effects.

Beyond Th1 response, Th2 immune response is also crucial to infection evolution, since it inhibits the mechanisms of *Leishmania* sp. death that are induced by macrophages, leading to disease progression. Th2 cellular response is promoted by IL-10, IL-4, and IL-5 cytokines that are known as downregulators of the immune response [2], and patients infected with *L.* (*L.*) *amazonensis* presented high levels of IL-10, IL-4, and TNF-*α* [42]. In the present study, *Leishmania* infection induced TNF-*α* and IL-10 expression strongly, but *C. brasiliense* subverted these cytokines, mainly after infection. IL-10 is the main regulator cytokine of Th1 type cellular immune response and acts as a inhibitor of IL-12, IL-2, TNF-*α*, and IFN-*γ* synthesis, favoring the parasite persistence [43]. Thus, the results of the present study suggest that the hydroalcoholic extract of the studied plant species, under these conditions, provides an immunomodulatory capacity to inhibit or reduce the mRNA expression of cytokine related to Th2 cell and inflammation, favoring the leishmaniasis resolution.

IL-1*β* expression was also downregulated by *C. brasiliense*. First, following inoculation of parasites in the skin and the start of phagocytosis of promastigote forms, it activated mononuclear phagocytes releasing TNF-*α* and IL-1*β* cytokines, acting in innate immunity and inflammation, for the purpose of stimulating the recruitment of neutrophils and monocytes to sites of infection. These actions occur through the increased expression of adhesion molecules on the vascular endothelium (ligands for selectins and integrins) and by inducing chemotaxis of leukocytes [2]. Also, the inflammasome-derived IL-1*β* induces the resistance to *Leishmania amazonensis* in infected macrophages [44]. Thus, IL-1*β* can promote the proliferation and activation of Th2 cells and suppress the Th1 response [45]. Therefore, *C. brasiliense* is a potential IL-1*β* inhibitor, favoring the leishmaniasis resolution.

In short, our data suggest that the hydroalcoholic extract of *C. brasiliense* at 30 *μ*g/mL has leishmanicidal activity and immunomodulatory effects. This plant modulated the immune response in murine macrophages infected with *L.* (*L.*) *amazonensis* or used alone, due to inhibiting completely or reducing significantly mRNA expression of cytokines related to Th1 and Th2 cellular response. Therefore, we suggest that the other inflammatory mediators, such as prostaglandins and leukotrienes, are investigated in animal models submitted to *C. brasiliense* and *Leishmania* infection. The cytokine inhibition profile induced by *C. brasiliense* indicated that this plant has an anti-inflammatory activity. Thus, the antileishmanial and anti-inflammatory immunomodulatory effects showed the potential of this plant as an alternative therapy to cutaneous leishmaniasis caused by *L.* (*L.*) *amazonensis*.

## Figures and Tables

**Table 1:** Gene and PCR conditions.

Gene	Reference	Annealing temperature (°C)	Cycles	Fragments (bp)
TNF-*α*	Kolodziej et al. [31]	61	36	276
IL-1*β*	Chen et al. [32]	57	30	204
IL-12	Kolodziej et al. [31]	61	36	312
IL-18	Kolodziej et al. [31]	61	36	440
IFN-*γ*	Chen et al. [32]	56	35	336
iNOS	Chen et al. [32]	55	30	311
IL-10	Kolodziej et al. [31]	61	36	256
GAPDH	Byrne et al. [33]	60	35	239

GAPDH: glyceraldehyde-3 phosphate dehydrogenase; IFN-*γ*: interferon-*γ*; IL: interleukin; iNOS: inducible nitric oxide synthase; TNF-*α*: tumour necrosis factor *α*.
